# Methyl 1-methyl-3-phenyl-1,2,3,3a,4,9b-hexa­hydro­benzo[*f*]chromeno[4,3-*b*]pyrrole-3a-carboxyl­ate

**DOI:** 10.1107/S1600536809012914

**Published:** 2009-04-10

**Authors:** B. Gunasekaran, S. Kathiravan, R. Raghunathan, V. Renuga, V. Manivannan

**Affiliations:** a Department of Physics, AMET University, Kanathur, Chennai 603 112, India; b Department of Organic Chemistry, University of Madras, Guindy Campus, Chennai 600 025, India; c Department of Chemistry, National College, Thiruchirapalli, Tamil Nadu, India; d Department of Research and Development, PRIST University, Vallam, Thanjavur 613 403, Tamil Nadu, India

## Abstract

In the title compound, C_24_H_23_NO_3_, the dihedral angle between the naphthalene ring system and the phenyl ring is 76.82 (6)°. The pyrrolidine ring adopts an envelope conformation. In the crystal, weak inter­molecular C—H⋯O and C—H⋯π inter­actions are observed.

## Related literature

For the biological activity of chromenopyrrole, see: Caine (1993 ▶); Tidey (1992 ▶); Carlson (1993 ▶); Sokoloff *et al.* (1990 ▶); Wilner (1985 ▶); Sobral & Rocha Gonsalves (2001*a* ▶,*b* ▶); Brockmann & Tour (1995 ▶); Suslick *et al.* (1992 ▶); Di Natale *et al.* (1998 ▶). For a related structure, see: Nirmala *et al.* (2008 ▶). For graph-set notation, see: Bernstein *et al.* (1995 ▶).
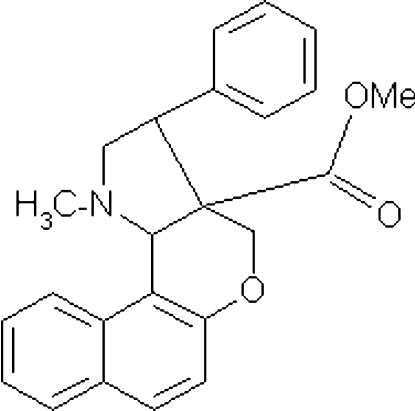


## Experimental

### 

#### Crystal data

C_24_H_23_NO_3_
*M* *_r_* = 373.43
Monoclinic, 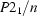

*a* = 13.2332 (6) Å
*b* = 10.3574 (4) Å
*c* = 15.0865 (6) Å
β = 111.530 (2)°
*V* = 1923.50 (14) Å^3^
*Z* = 4
Mo *K*α radiation
μ = 0.09 mm^−1^
*T* = 293 K
0.25 × 0.20 × 0.15 mm

#### Data collection

Bruker Kappa APEX2 CCD diffractometer
Absorption correction: multi-scan (**SADABS**; Sheldrick, 1996 ▶) *T*_min_ = 0.979, *T*_max_ = 0.987
19089 measured reflections
3499 independent reflections
2413 reflections with *I* > 2σ(*I*)
*R*_int_ = 0.041

#### Refinement

*R*[*F* ^2^ > 2σ(*F* ^2^)] = 0.040
*wR*(*F* ^2^) = 0.107
*S* = 1.02
3499 reflections
255 parameters
H-atom parameters constrained
Δρ_max_ = 0.14 e Å^−3^
Δρ_min_ = −0.14 e Å^−3^

### 

Data collection: *APEX2* (Bruker, 2004 ▶); cell refinement: *SAINT* (Bruker, 2004 ▶); data reduction: *SAINT*; program(s) used to solve structure: *SHELXS97* (Sheldrick, 2008 ▶); program(s) used to refine structure: *SHELXL97* (Sheldrick, 2008 ▶); molecular graphics: *PLATON* (Spek, 2009 ▶); software used to prepare material for publication: *SHELXL97*.

## Supplementary Material

Crystal structure: contains datablocks global, I. DOI: 10.1107/S1600536809012914/is2404sup1.cif

Structure factors: contains datablocks I. DOI: 10.1107/S1600536809012914/is2404Isup2.hkl

Additional supplementary materials:  crystallographic information; 3D view; checkCIF report

## Figures and Tables

**Table 1:** Hydrogen-bond geometry (Å, °)

*D*—H⋯*A*	*D*—H	H⋯*A*	*D*⋯*A*	*D*—H⋯*A*
C15—H15⋯O2^i^	0.98	2.53	3.347 (2)	141
C16—H16*C*⋯*Cg*^ii^	0.96	2.79	3.689 (4)	156

Symmetry codes: (i) 
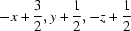
; (ii) 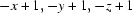. *Cg* is the centroid of the C4–C9 ring.

**Fig. 1.**
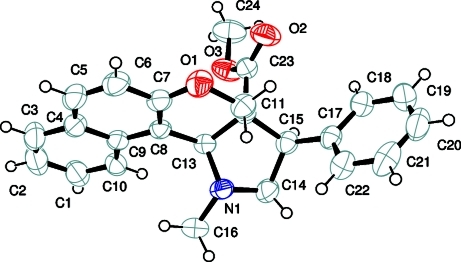
The molecular structure of (I), with atom labels and 50% probability displacement ellipsoids for non-H atoms.

**Fig. 2.**
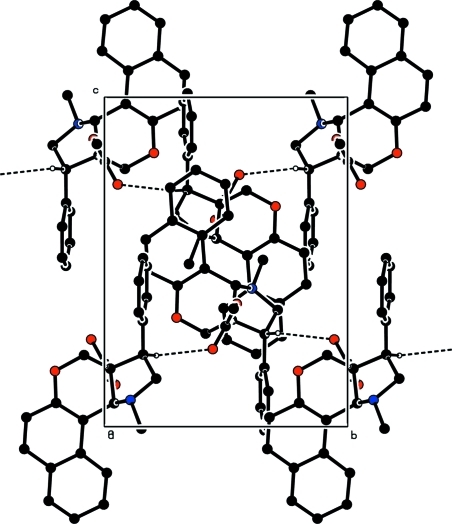
The packing of (I), viewed down the a axis. Hydrogen bonds are shown as dashed lines.

## supplementary crystallographic information

### Comment

Chromenopyrrole compounds are used in the treatment of impulsive disorders (Caine, 1993), aggressiveness (Tidey, 1992), parkinson's disease (Carlson, 1993), psychoses, memory disorders (Sokoloff *et al.*, 1990), anxiety and depression (Wilner, 1985). Pyrroles are also very useful precursors in porphyrin synthesis (Sobral & Rocha Gonsalves, 2001*a*,*b*), and as monomers for polymer chemistry (Brockmann & Tour, 1995), with applications ranging from nonlinear optical materials (Suslick *et al.*, 1992) to electronic noses (Di Natale *et al.*, 1998).

The geometric parameters of the title molecule (Fig. 1) agree well with reported similar structure (Nirmala *et al.*, 2008). The six-membered heterocyclic ring [C7/O1/C11/C12/C13/C8] of the benzochromenopyrrole moiety adopts a half-chair conformation [O1—C7—C8—C13 = 0.5 (2)° and O1—C11—C12—C13 = -60.09 (17)°]. The sum of bond angles around N1 [332.37 (14)°] indicates the *sp*^3^ hybridized state of atom N1 in the molecule. The C16—N1—C13—C12 torsion angle is -173.40 (14)°, which corresponds to an antiperiplanar conformation.

The crystal packing is stabilized by weak intramolecular C—H···O and C—H···N interactions. In addition, the structure is stabilized by weak intermolecular C—H···O and C—H···π (C16—H16C···*Cg*; *Cg* is the centroid of ring defined by the atoms C4–C9) interactions (Table 1). The C11—H11A···N1 and C13—H13···O3 interactions each generate an S(5) graph set motif (Bernstein *et al.*, 1995).

### Experimental

A mixture of (*z*)-methyl-5-(1-formylnathalen-2-yl-)-3-phenylpent -2-enoate and sarcosine were refluxed in benzene for 20 h and the solvent was removed under reduced pressure. The crude product was subjected to column chromatography to get the pure product.

### Refinement

H atoms were positioned geometrically and refined using riding model, with C—H = 0.93 Å and *U*_iso_(H) = 1.2*U*_eq_(C) for aromatic C—H, C—H = 0.98 Å and *U*_iso_(H) = 1.2*U*_eq_(C) for C—H, C—H = 0.97 Å and *U*_iso_(H) = 1.2*U*_eq_(C) for CH_2_, and C—H = 0.96 Å and *U*_iso_(H) = 1.5*U*_eq_(C) for CH_3_.

### Crystal data

**Table:**

C_24_H_23_NO_3_	*F*(000) = 792
*M**_r_* = 373.43	*D*_x_ = 1.290 Mg m^−^^3^
Monoclinic, *P*2_1_/*n*	Mo *K*α radiation, λ = 0.71073 Å
Hall symbol: -P 2yn	Cell parameters from 3865 reflections
*a* = 13.2332 (6) Å	θ = 1.8–25.3°
*b* = 10.3574 (4) Å	µ = 0.09 mm^−^^1^
*c* = 15.0865 (6) Å	*T* = 293 K
β = 111.530 (2)°	Block, colourless
*V* = 1923.50 (14) Å^3^	0.25 × 0.20 × 0.15 mm
*Z* = 4	

### Data collection

**Table:**

Bruker Kappa APEX2 CCD diffractometer	3499 independent reflections
Radiation source: fine-focus sealed tube	2413 reflections with *I* > 2σ(*I*)
graphite	*R*_int_ = 0.041
Detector resolution: 0 pixels mm^-1^	θ_max_ = 25.3°, θ_min_ = 2.4°
ω and φ scans	*h* = −15→15
Absorption correction: multi-scan (*SADABS*; Sheldrick, 1996)	*k* = −11→12
*T*_min_ = 0.979, *T*_max_ = 0.987	*l* = −18→18
19089 measured reflections	

### Refinement

**Table:**

Refinement on *F*^2^	Primary atom site location: structure-invariant direct methods
Least-squares matrix: full	Secondary atom site location: difference Fourier map
*R*[*F*^2^ > 2σ(*F*^2^)] = 0.040	Hydrogen site location: inferred from neighbouring sites
*wR*(*F*^2^) = 0.107	H-atom parameters constrained
*S* = 1.02	*w* = 1/[σ^2^(*F*_o_^2^) + (0.0458*P*)^2^ + 0.3587*P*] where *P* = (*F*_o_^2^ + 2*F*_c_^2^)/3
3499 reflections	(Δ/σ)_max_ < 0.001
255 parameters	Δρ_max_ = 0.14 e Å^−^^3^
0 restraints	Δρ_min_ = −0.14 e Å^−^^3^

### Special details

**Table:**

Geometry. All e.s.d.'s (except the e.s.d. in the dihedral angle between two l.s. planes) are estimated using the full covariance matrix. The cell e.s.d.'s are taken into account individually in the estimation of e.s.d.'s in distances, angles and torsion angles; correlations between e.s.d.'s in cell parameters are only used when they are defined by crystal symmetry. An approximate (isotropic) treatment of cell e.s.d.'s is used for estimating e.s.d.'s involving l.s. planes.
Refinement. Refinement of *F*^2^ against ALL reflections. The weighted *R*-factor *wR* and goodness of fit *S* are based on *F*^2^, conventional *R*-factors *R* are based on *F*, with *F* set to zero for negative *F*^2^. The threshold expression of *F*^2^ > σ(*F*^2^) is used only for calculating *R*-factors(gt) *etc*. and is not relevant to the choice of reflections for refinement. *R*-factors based on *F*^2^ are statistically about twice as large as those based on *F*, and *R*- factors based on ALL data will be even larger.

### Fractional atomic coordinates and isotropic or equivalent isotropic displacement parameters (Å^2^)

**Table:**

	*x*	*y*	*z*	*U*_iso_*/*U*_eq_	
O1	0.87620 (10)	0.79656 (11)	0.16896 (8)	0.0560 (3)	
O3	0.64358 (10)	1.04425 (13)	0.12731 (9)	0.0610 (4)	
C8	0.80155 (13)	0.91239 (15)	0.02018 (11)	0.0404 (4)	
O2	0.72839 (11)	0.94550 (14)	0.26458 (9)	0.0684 (4)	
C7	0.83173 (14)	0.80090 (16)	0.07191 (12)	0.0473 (4)	
N1	0.91939 (11)	1.10692 (13)	0.08087 (9)	0.0452 (4)	
C13	0.81799 (13)	1.03987 (15)	0.07102 (10)	0.0388 (4)	
H13	0.7558	1.0965	0.0393	0.047*	
C9	0.74815 (13)	0.90220 (16)	−0.08105 (12)	0.0452 (4)	
C11	0.91380 (14)	0.91616 (15)	0.21733 (12)	0.0483 (4)	
H11A	0.9827	0.9387	0.2124	0.058*	
H11B	0.9258	0.9059	0.2843	0.058*	
C10	0.70597 (14)	1.00976 (19)	−0.14052 (12)	0.0508 (4)	
H10	0.7133	1.0915	−0.1134	0.061*	
C4	0.73409 (15)	0.77937 (18)	−0.12524 (13)	0.0553 (5)	
C12	0.83340 (13)	1.02418 (15)	0.17618 (10)	0.0394 (4)	
C23	0.73092 (14)	0.99806 (16)	0.19506 (11)	0.0439 (4)	
C6	0.81600 (17)	0.67864 (17)	0.02815 (15)	0.0605 (5)	
H6	0.8373	0.6044	0.0650	0.073*	
C17	0.95781 (14)	1.15779 (15)	0.32102 (11)	0.0440 (4)	
C15	0.88052 (14)	1.15911 (15)	0.21892 (11)	0.0448 (4)	
H15	0.8187	1.2134	0.2160	0.054*	
C5	0.76969 (17)	0.66982 (19)	−0.06782 (15)	0.0665 (6)	
H5	0.7613	0.5889	−0.0964	0.080*	
C16	0.93000 (15)	1.15213 (18)	−0.00619 (12)	0.0555 (5)	
H16A	0.8733	1.2130	−0.0371	0.083*	
H16B	0.9244	1.0802	−0.0479	0.083*	
H16C	0.9993	1.1931	0.0087	0.083*	
C3	0.68054 (18)	0.7705 (2)	−0.22541 (15)	0.0715 (6)	
H3	0.6717	0.6900	−0.2546	0.086*	
C22	1.06811 (15)	1.14119 (17)	0.34720 (13)	0.0580 (5)	
H22	1.0977	1.1295	0.3006	0.070*	
C1	0.65478 (16)	0.9971 (2)	−0.23651 (13)	0.0629 (5)	
H1	0.6280	1.0699	−0.2738	0.076*	
C2	0.64222 (18)	0.8763 (3)	−0.27927 (15)	0.0753 (6)	
H2	0.6075	0.8685	−0.3449	0.090*	
C14	0.92485 (17)	1.21276 (17)	0.14582 (12)	0.0580 (5)	
H14A	0.8811	1.2850	0.1117	0.070*	
H14B	0.9992	1.2417	0.1771	0.070*	
C18	0.91714 (17)	1.17418 (17)	0.39259 (12)	0.0571 (5)	
H18	0.8428	1.1855	0.3765	0.069*	
C19	0.9842 (2)	1.1741 (2)	0.48683 (14)	0.0760 (7)	
H19	0.9550	1.1846	0.5338	0.091*	
C21	1.13528 (18)	1.14174 (19)	0.44215 (15)	0.0725 (6)	
H21	1.2097	1.1306	0.4588	0.087*	
C20	1.0936 (2)	1.1585 (2)	0.51178 (15)	0.0784 (7)	
H20	1.1392	1.1594	0.5756	0.094*	
C24	0.54215 (16)	1.0313 (2)	0.14187 (16)	0.0749 (6)	
H24A	0.5298	0.9421	0.1518	0.112*	
H24B	0.4839	1.0628	0.0868	0.112*	
H24C	0.5453	1.0806	0.1967	0.112*	

### Atomic displacement parameters (Å^2^)

**Table:**

	*U*^11^	*U*^22^	*U*^33^	*U*^12^	*U*^13^	*U*^23^
O1	0.0678 (9)	0.0358 (7)	0.0620 (8)	0.0033 (6)	0.0211 (6)	0.0046 (6)
O3	0.0449 (7)	0.0785 (9)	0.0673 (8)	0.0114 (7)	0.0296 (6)	0.0219 (7)
C8	0.0387 (9)	0.0373 (9)	0.0525 (10)	−0.0033 (7)	0.0254 (8)	−0.0041 (8)
O2	0.0685 (9)	0.0824 (10)	0.0587 (8)	−0.0099 (7)	0.0284 (7)	0.0197 (7)
C7	0.0473 (11)	0.0413 (10)	0.0600 (11)	−0.0024 (8)	0.0278 (9)	−0.0016 (8)
N1	0.0440 (9)	0.0439 (8)	0.0500 (8)	−0.0079 (6)	0.0199 (6)	0.0019 (6)
C13	0.0379 (9)	0.0367 (9)	0.0441 (9)	0.0004 (7)	0.0180 (7)	0.0023 (7)
C9	0.0407 (10)	0.0490 (10)	0.0540 (10)	−0.0075 (8)	0.0270 (8)	−0.0092 (8)
C11	0.0501 (11)	0.0396 (10)	0.0527 (10)	0.0019 (8)	0.0159 (8)	0.0033 (8)
C10	0.0472 (10)	0.0607 (12)	0.0481 (10)	−0.0038 (9)	0.0217 (8)	−0.0073 (9)
C4	0.0563 (12)	0.0537 (12)	0.0659 (12)	−0.0100 (9)	0.0343 (10)	−0.0151 (10)
C12	0.0400 (9)	0.0372 (9)	0.0411 (8)	0.0006 (7)	0.0151 (7)	0.0032 (7)
C23	0.0505 (11)	0.0385 (9)	0.0441 (9)	−0.0032 (8)	0.0190 (8)	−0.0006 (8)
C6	0.0731 (14)	0.0364 (10)	0.0847 (14)	−0.0042 (9)	0.0437 (11)	−0.0044 (9)
C17	0.0488 (11)	0.0331 (9)	0.0479 (9)	−0.0020 (8)	0.0150 (8)	−0.0029 (7)
C15	0.0481 (10)	0.0374 (9)	0.0470 (9)	0.0000 (8)	0.0151 (8)	−0.0005 (7)
C5	0.0817 (15)	0.0477 (12)	0.0858 (15)	−0.0156 (11)	0.0492 (12)	−0.0239 (11)
C16	0.0533 (12)	0.0587 (12)	0.0626 (11)	−0.0069 (9)	0.0310 (9)	0.0072 (9)
C3	0.0717 (15)	0.0794 (16)	0.0716 (14)	−0.0169 (12)	0.0357 (12)	−0.0354 (12)
C22	0.0532 (12)	0.0536 (12)	0.0621 (12)	−0.0017 (9)	0.0151 (9)	−0.0105 (9)
C1	0.0578 (12)	0.0812 (15)	0.0518 (11)	0.0006 (10)	0.0225 (9)	−0.0056 (10)
C2	0.0688 (15)	0.1018 (19)	0.0555 (12)	−0.0036 (13)	0.0228 (11)	−0.0226 (13)
C14	0.0734 (14)	0.0471 (11)	0.0495 (10)	−0.0171 (9)	0.0178 (9)	−0.0005 (8)
C18	0.0661 (13)	0.0527 (11)	0.0553 (11)	−0.0083 (9)	0.0255 (10)	−0.0086 (9)
C19	0.104 (2)	0.0731 (15)	0.0519 (12)	−0.0254 (13)	0.0300 (13)	−0.0113 (10)
C21	0.0602 (14)	0.0545 (13)	0.0789 (15)	−0.0040 (10)	−0.0026 (12)	−0.0073 (11)
C20	0.103 (2)	0.0590 (14)	0.0502 (12)	−0.0214 (13)	0.0010 (13)	−0.0019 (10)
C24	0.0524 (13)	0.0869 (16)	0.1010 (16)	0.0095 (11)	0.0467 (12)	0.0171 (13)

### Geometric parameters (Å, °)

**Table:**

O1—C7	1.3637 (19)	C17—C18	1.382 (2)
O1—C11	1.4311 (19)	C17—C15	1.504 (2)
O3—C23	1.320 (2)	C15—C14	1.531 (2)
O3—C24	1.444 (2)	C15—H15	0.9800
C8—C7	1.369 (2)	C5—H5	0.9300
C8—C9	1.432 (2)	C16—H16A	0.9600
C8—C13	1.502 (2)	C16—H16B	0.9600
O2—C23	1.1931 (18)	C16—H16C	0.9600
C7—C6	1.408 (2)	C3—C2	1.347 (3)
N1—C16	1.449 (2)	C3—H3	0.9300
N1—C14	1.454 (2)	C22—C21	1.381 (3)
N1—C13	1.469 (2)	C22—H22	0.9300
C13—C12	1.532 (2)	C1—C2	1.389 (3)
C13—H13	0.9800	C1—H1	0.9300
C9—C10	1.411 (2)	C2—H2	0.9300
C9—C4	1.417 (2)	C14—H14A	0.9700
C11—C12	1.511 (2)	C14—H14B	0.9700
C11—H11A	0.9700	C18—C19	1.372 (3)
C11—H11B	0.9700	C18—H18	0.9300
C10—C1	1.361 (2)	C19—C20	1.364 (3)
C10—H10	0.9300	C19—H19	0.9300
C4—C5	1.400 (3)	C21—C20	1.365 (3)
C4—C3	1.417 (3)	C21—H21	0.9300
C12—C23	1.509 (2)	C20—H20	0.9300
C12—C15	1.569 (2)	C24—H24A	0.9600
C6—C5	1.352 (3)	C24—H24B	0.9600
C6—H6	0.9300	C24—H24C	0.9600
C17—C22	1.376 (2)		
			
C7—O1—C11	116.82 (12)	C14—C15—C12	103.10 (12)
C23—O3—C24	116.58 (14)	C17—C15—H15	107.1
C7—C8—C9	118.20 (15)	C14—C15—H15	107.1
C7—C8—C13	119.55 (14)	C12—C15—H15	107.1
C9—C8—C13	122.11 (14)	C6—C5—C4	121.67 (17)
O1—C7—C8	124.11 (15)	C6—C5—H5	119.2
O1—C7—C6	113.89 (16)	C4—C5—H5	119.2
C8—C7—C6	121.97 (16)	N1—C16—H16A	109.5
C16—N1—C14	111.70 (13)	N1—C16—H16B	109.5
C16—N1—C13	116.73 (13)	H16A—C16—H16B	109.5
C14—N1—C13	104.01 (13)	N1—C16—H16C	109.5
N1—C13—C8	115.02 (13)	H16A—C16—H16C	109.5
N1—C13—C12	100.13 (12)	H16B—C16—H16C	109.5
C8—C13—C12	112.01 (13)	C2—C3—C4	121.4 (2)
N1—C13—H13	109.8	C2—C3—H3	119.3
C8—C13—H13	109.8	C4—C3—H3	119.3
C12—C13—H13	109.8	C17—C22—C21	120.56 (19)
C10—C9—C4	117.20 (15)	C17—C22—H22	119.7
C10—C9—C8	123.04 (15)	C21—C22—H22	119.7
C4—C9—C8	119.75 (16)	C10—C1—C2	120.6 (2)
O1—C11—C12	111.76 (13)	C10—C1—H1	119.7
O1—C11—H11A	109.3	C2—C1—H1	119.7
C12—C11—H11A	109.3	C3—C2—C1	119.85 (19)
O1—C11—H11B	109.3	C3—C2—H2	120.1
C12—C11—H11B	109.3	C1—C2—H2	120.1
H11A—C11—H11B	107.9	N1—C14—C15	105.93 (13)
C1—C10—C9	121.78 (18)	N1—C14—H14A	110.6
C1—C10—H10	119.1	C15—C14—H14A	110.6
C9—C10—H10	119.1	N1—C14—H14B	110.6
C5—C4—C9	118.71 (17)	C15—C14—H14B	110.6
C5—C4—C3	122.04 (19)	H14A—C14—H14B	108.7
C9—C4—C3	119.19 (19)	C19—C18—C17	121.3 (2)
C23—C12—C11	109.62 (13)	C19—C18—H18	119.3
C23—C12—C13	115.29 (13)	C17—C18—H18	119.3
C11—C12—C13	108.05 (13)	C20—C19—C18	120.2 (2)
C23—C12—C15	109.34 (13)	C20—C19—H19	119.9
C11—C12—C15	112.14 (13)	C18—C19—H19	119.9
C13—C12—C15	102.29 (12)	C20—C21—C22	120.7 (2)
O2—C23—O3	123.16 (16)	C20—C21—H21	119.7
O2—C23—C12	124.39 (16)	C22—C21—H21	119.7
O3—C23—C12	112.40 (13)	C19—C20—C21	119.35 (19)
C5—C6—C7	119.59 (18)	C19—C20—H20	120.3
C5—C6—H6	120.2	C21—C20—H20	120.3
C7—C6—H6	120.2	O3—C24—H24A	109.5
C22—C17—C18	117.86 (16)	O3—C24—H24B	109.5
C22—C17—C15	123.11 (16)	H24A—C24—H24B	109.5
C18—C17—C15	119.03 (16)	O3—C24—H24C	109.5
C17—C15—C14	116.40 (15)	H24A—C24—H24C	109.5
C17—C15—C12	115.53 (13)	H24B—C24—H24C	109.5
			
C11—O1—C7—C8	−13.5 (2)	C13—C12—C23—O2	155.96 (16)
C11—O1—C7—C6	168.25 (15)	C15—C12—C23—O2	−89.50 (19)
C9—C8—C7—O1	−175.27 (14)	C11—C12—C23—O3	−148.53 (14)
C13—C8—C7—O1	0.5 (2)	C13—C12—C23—O3	−26.37 (19)
C9—C8—C7—C6	2.8 (2)	C15—C12—C23—O3	88.17 (16)
C13—C8—C7—C6	178.56 (16)	O1—C7—C6—C5	178.20 (16)
C16—N1—C13—C8	66.33 (19)	C8—C7—C6—C5	−0.1 (3)
C14—N1—C13—C8	−170.14 (13)	C22—C17—C15—C14	−31.9 (2)
C16—N1—C13—C12	−173.44 (13)	C18—C17—C15—C14	148.01 (16)
C14—N1—C13—C12	−49.91 (15)	C22—C17—C15—C12	89.3 (2)
C7—C8—C13—N1	94.95 (17)	C18—C17—C15—C12	−90.82 (19)
C9—C8—C13—N1	−89.46 (18)	C23—C12—C15—C17	88.85 (17)
C7—C8—C13—C12	−18.5 (2)	C11—C12—C15—C17	−32.95 (19)
C9—C8—C13—C12	157.09 (14)	C13—C12—C15—C17	−148.48 (14)
C7—C8—C9—C10	174.89 (15)	C23—C12—C15—C14	−143.05 (14)
C13—C8—C9—C10	−0.8 (2)	C11—C12—C15—C14	95.15 (16)
C7—C8—C9—C4	−3.9 (2)	C13—C12—C15—C14	−20.38 (16)
C13—C8—C9—C4	−179.52 (14)	C7—C6—C5—C4	−1.7 (3)
C7—O1—C11—C12	44.22 (19)	C9—C4—C5—C6	0.5 (3)
C4—C9—C10—C1	−0.7 (2)	C3—C4—C5—C6	−176.74 (18)
C8—C9—C10—C1	−179.46 (15)	C5—C4—C3—C2	176.87 (19)
C10—C9—C4—C5	−176.58 (16)	C9—C4—C3—C2	−0.4 (3)
C8—C9—C4—C5	2.3 (2)	C18—C17—C22—C21	−0.3 (3)
C10—C9—C4—C3	0.8 (2)	C15—C17—C22—C21	179.54 (17)
C8—C9—C4—C3	179.63 (15)	C9—C10—C1—C2	0.1 (3)
O1—C11—C12—C23	66.29 (17)	C4—C3—C2—C1	−0.1 (3)
O1—C11—C12—C13	−60.09 (17)	C10—C1—C2—C3	0.3 (3)
O1—C11—C12—C15	−172.07 (13)	C16—N1—C14—C15	164.03 (14)
N1—C13—C12—C23	160.94 (13)	C13—N1—C14—C15	37.29 (17)
C8—C13—C12—C23	−76.68 (17)	C17—C15—C14—N1	118.28 (16)
N1—C13—C12—C11	−76.07 (15)	C12—C15—C14—N1	−9.28 (17)
C8—C13—C12—C11	46.31 (17)	C22—C17—C18—C19	0.0 (3)
N1—C13—C12—C15	42.39 (15)	C15—C17—C18—C19	−179.84 (17)
C8—C13—C12—C15	164.78 (13)	C17—C18—C19—C20	0.5 (3)
C24—O3—C23—O2	1.5 (3)	C17—C22—C21—C20	0.1 (3)
C24—O3—C23—C12	−176.18 (15)	C18—C19—C20—C21	−0.7 (3)
C11—C12—C23—O2	33.8 (2)	C22—C21—C20—C19	0.4 (3)

### Hydrogen-bond geometry (Å, °)

**Table:**

*D*—H···*A*	*D*—H	H···*A*	*D*···*A*	*D*—H···*A*
C11—H11A···N1	0.97	2.54	2.874 (2)	100
C13—H13···O3	0.98	2.39	2.7366 (19)	100
C15—H15···O2^i^	0.98	2.53	3.347 (2)	141
C16—H16C···Cg^ii^	0.96	2.79	3.689 (4)	156

Symmetry codes: (i) −*x*+3/2, *y*+1/2, −*z*+1/2; (ii) −*x*+1, −*y*+1, −*z*+1.
